# From Discovery to Translation: Characterization of C-Mannosyltryptophan and Pseudouridine as Markers of Kidney Function

**DOI:** 10.1038/s41598-017-17107-5

**Published:** 2017-12-12

**Authors:** Peggy Sekula, Katja Dettmer, Franziska C. Vogl, Wolfram Gronwald, Lisa Ellmann, Robert P. Mohney, Kai-Uwe Eckardt, Karsten Suhre, Gabi Kastenmüller, Peter J. Oefner, Anna Köttgen

**Affiliations:** 1 Institute of Genetic Epidemiology, Faculty of Medicine and Medical Center – University of Freiburg, Hugstetter Str. 49, 79106 Freiburg, Germany; 2 Institute of Functional Genomics, University of Regensburg, Am BioPark 9, 93053 Regensburg, Germany; 3 Metabolon, Inc., Durham, NC USA; 4 Department of Nephrology and Hypertension, University of Erlangen-Nürnberg, 91054 Erlangen, Germany; 5 Department of Physiology and Biophysics, Weill Cornell Medicine - Qatar, PO 24144 Doha, Qatar; 6 Institute of Bioinformatics and Systems Biology, Helmholtz Zentrum München – German Research Center for Environmental Health, 85764 Neuherberg, Germany; 7 German Center for Diabetes Research (DZD), 85764 Neuherberg, Germany; 8 Department of Twin Research and Genetic Epidemiology, Kings College London, London, United Kingdom

## Abstract

Using a non-targeted metabolomics platform, we recently identified C-mannosyltryptophan and pseudouridine as non-traditional kidney function markers. The aims of this study were to obtain absolute concentrations of both metabolites in blood and urine from individuals with and without CKD to provide reference ranges and to assess their fractional excretions (FE), and to assess the agreement with their non-targeted counterparts. In individuals without/with CKD, mean plasma and urine concentrations for C-mannosyltryptophan were 0.26/0.72 µmol/L and 3.39/4.30 µmol/mmol creatinine, respectively. The respective concentrations for pseudouridine were 2.89/5.67 µmol/L and 39.7/33.9 µmol/mmol creatinine. Median (25^th^, 75^th^ percentiles) FEs were 70.8% (65.6%, 77.8%) for C-mannosyltryptophan and 76.0% (68.6%, 82.4%) for pseudouridine, indicating partial net reabsorption. Association analyses validated reported associations between single metabolites and eGFR. Targeted measurements of both metabolites agreed well with the non-targeted measurements, especially in urine. Agreement for composite nephrological measures FE and urinary metabolite-to-creatinine ratio was lower, but could be improved by replacing non-targeted creatinine measurements with a standard clinical creatinine test. In summary, targeted quantification and additional characterization in relevant populations are necessary steps in the translation of non-traditional biomarkers in nephrology from non-targeted discovery to clinical application.

## Introduction

Chronic kidney disease (CKD) is a major public health concern affecting approximately 10% of the population^1^. It is defined based on measures of kidney damage and kidney function. To assess kidney function, metabolites such as creatinine and urea are measured in blood and used to estimate the glomerular filtration rate (GFR)^2,3^. Currently used markers of kidney function, however, have limitations. For example, plasma creatinine concentrations only exceed the normal range when more than 50% of kidney function has been lost and also depend on age, sex and race as proxies of muscle mass^4^. The identification of additional kidney function markers is therefore of clinical importance in the diagnosis, staging and monitoring of CKD.

Recently, we conducted a study in population-based cohorts using a non-targeted metabolomics approach for metabolite quantification to discover and replicate serum metabolites that may be useful kidney function markers beyond creatinine^5^. We identified C-mannosyltryptophan and pseudouridine as strong correlates of estimated and measured GFR. Our study confirmed previous findings using similar non-targeted approaches^6,7^ and showed that, in contrast to serum creatinine, these potential alternative or complementary markers of kidney function showed little dependency on sex.

While non-targeted metabolomics methods have the advantage of detecting a broad spectrum of metabolites from different metabolic pathways, including metabolites of yet unknown identity^8^, they do not necessarily deliver absolute and accurate metabolite concentrations. This complicates the comparison of metabolite levels across populations and across the two biofluids of primary interest in nephrology, namely blood and urine. In our previous project, C-mannosyltryptophan and pseudouridine were identified based on non-targeted gas and liquid chromatography coupled to (nominal mass) mass spectrometry (GC/MS, LC/MS). Actual molar concentration ranges of C-mannosyltryptophan and pseudouridine in blood and urine, as well as their fractional excretions (FE), were not described.

**Figure 1:**
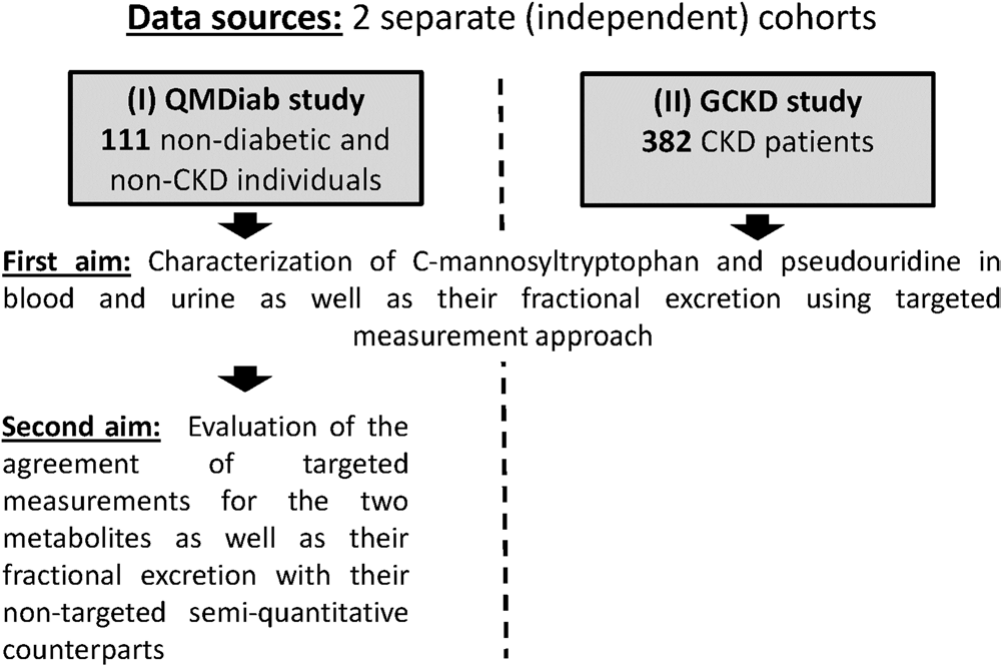
Schematic representation of the project’s data sources and aims.

On the way to clinical implementation, however, detailed knowledge about these two non-traditional markers in both healthy individuals and CKD patients is important. The first aim of the current study was to fill this gap by using a targeted approach to quantify C-mannosyltryptophan and pseudouridine employing liquid chromatography-triple quadrupole mass spectrometry in combination with stable isotope-labeled internal standards. The second aim was to systematically evaluate the agreement of non-targeted semi-quantitative and targeted absolute measurements for the two markers with special emphasis on composite measures of nephrological interest, the urinary metabolite-to-creatinine ratio and the FE. A schematic presentation of the project is provided in Fig. 1.

## Results

### Concentrations and fractional excretion of C-mannosyltryptophan and pseudouridine

Targeted measurements of C-mannosyltryptophan and pseudouridine were obtained for 111 control participants without diabetes and CKD from the QMDiab Study and for 382 CKD patients selected from the GCKD study (Table 1).

**Table 1:** Description of study populations.

Study		QMDiab	GCKD
Health status		Non-CKD individuals	CKD patients
Ancestry		Arab/Asian	European
Population size	N	111	382
Sex, male	N (%)	46 (41%)	235 (62%)
Age, years	Mean (SD)	38.3 (11.6)	59.5 (12.0)
Body mass index, kg/m²	Mean (SD)	28.3 (5.1)	30.1 (5.9)
Blood measures^*^			
- Creatinine, mg/dL	Mean (SD)	0.80 (0.18)	1.47 (0.43)
- Cystatin C, mg/L	Mean (SD)	NA	1.54 (0.42)
eGFR CKD-EPI, mL/min/1.73 m²			
- based on creatinine	Mean (SD)	104.0 (15.7)	50.9 (17.6)
- based on cystatin C	Mean (SD)	NA	47.6 (17.0)
Urine measures^*^			
- Creatinine, mg/dL	Mean (SD)	139.2 (84.4)	78.7 (52.9)
- UACR, mg/g	Median (IQR)	NA	62.5 (11.9–450.4)

^*^Creatinine in both blood (serum in GCKD and plasma for QMDiab) and urine were measured using a calibrated, enzymatic, standard clinical laboratory assay. Creatinine conversion: 1 mg/dL = 88.4 µmol/L. IQR – interquartile range; NA – not available.

**Figure 2:**
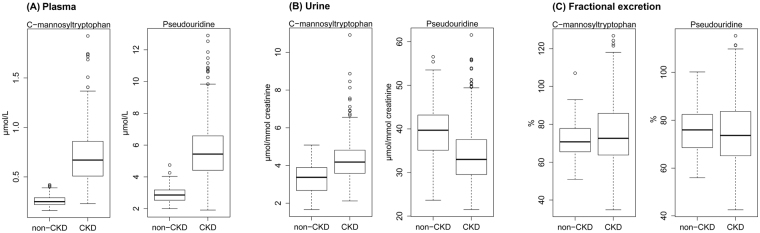
Distributions of targeted measurements of C-mannosyltryptophan and pseudouridine and their fractional excretion in individuals with and without CKD. (**A**) Plasma concentrations, (**B**) Urine concentrations, (**C**) Fractional excretion.

In the healthy QMDiab individuals, mean C-mannosyltryptophan concentrations were 0.26 µmol/L (SD: 0.05) in plasma and 3.39 µmol/mmol creatinine (SD: 0.80) in urine. Mean pseudouridine concentrations were 2.89 µmol/L (SD: 0.52) in plasma and 39.7 µmol/mmol creatinine (SD: 6.74) in urine (Table 2 and Fig. 2). The FE is an informative measure for kidney filtration markers, as it allows the assessment of a substance’s net secretion into urine or net reabsorption into the blood. The median (25^th^, 75^th^ percentiles) of the FE were 76.0% (68.6%, 82.4%) for pseudouridine and 70.8% (65.6%, 77.8%) for C-mannosyltryptophan, suggesting partial net reabsorption for both metabolites. Graphical illustration of the distributions of the plasma and urine concentrations of both markers as well as their FEs showed that a natural logarithmic transformation was appropriate in most cases to reduce skewness (Supplementary Fig. S1).

**Table 2:** Characterization of C-mannosyltryptophan and Pseudouridine.

Targeted LC/MS measurements	N	Mini-mum	Q1	Median	Q3	Maxi-mum	Mean(SD)
**Plasma (µmol/L)**							
C-mannosyltryptophan							
- Non-CKD individuals	111	0.16	0.22	0.25	0.29	0.42	0.26 (0.05)
- CKD patients	328	0.23	0.51	0.67	0.86	1.92	0.72 (0.28)
Pseudouridine							
- Non-CKD individuals	111	2.00	2.53	2.86	3.18	4.75	2.89 (0.52)
- CKD patients	329	1.91	4.41	5.44	6.59	12.9	5.67 (1.84)
**Urine (µmol/mmol creatinine)**							
C-mannosyltryptophan							
- Non-CKD individuals	111	1.66	2.67	3.37	3.89	5.08	3.39 (0.80)
- CKD patients	377	2.12	3.59	4.18	4.81	10.9	4.30 (1.05)
Pseudouridine							
- Non-CKD individuals	111	23.6	35.1	39.7	43.2	56.6	39.7 (6.74)
- CKD patients	377	21.5	29.5	33.0	37.5	61.6	33.9 (6.63)
**Fractional excretion (%)**							
C-mannosyltryptophan							
- Non-CKD individuals	111	51.0	65.6	70.8	77.8	107.0	71.8 (9.43)
- CKD patients	329	34.9	63.8	72.7	85.8	126.8	75.8 (17.4)
Pseudouridine							
- Non-CKD individuals	111	56.0	68.6	76.0	82.4	100.2	76.1 (9.66)
- CKD patients	329	42.5	65.2	73.7	83.7	115.4	74.6 (13.5)

Mean (SD) of targeted LC/MS measurements of creatinine (µmol/L): (a) Non-CKD individuals: plasma 56.5 (13.65), urine 11,220 (6,344.27), (b) CKD patients: plasma 124.3 (35.74), urine 6,399.0 (3,946.15).

In CKD patients, the mean plasma concentrations of C-mannosyltryptophan and pseudouridine were clearly higher than in the QMDiab controls (Table 2 and Fig. 2). In urine, mean absolute concentrations of both metabolites were lower in CKD patients than in healthy controls. When accounting for urine dilution, mean concentrations of C-mannosyltryptophan in urine were higher among CKD patients than controls, but lower for pseudouridine. While the shape of the metabolites’ distributions did not differ markedly between the two cohorts (Supplementary Fig. S1), the plasma concentrations and FEs had a wider range in CKD patients (Table 2).

In the healthy QMDiab individuals, we observed a strong positive correlation between the concentrations of the two metabolites within a given biofluid (Spearman correlation coefficient [SCC]: plasma 0.74, urine 0.81), while the correlations between biofluids were weak to moderate (SCC comparing plasma to urine: C-mannosyltryptophan 0.40, pseudouridine 0.20). Similar results were obtained in CKD patients (Table 3). Table 3 also shows correlations between targeted metabolite measurements in CKD patients and different established clinical laboratory measurements of kidney function markers, including a creatinine test that currently represents the clinical standard for the measurement of creatinine. For creatinine in blood, the SCC of targeted mass spectrometry-based plasma measurements and serum standard laboratory measurements was 0.96, showing excellent correlation. The same held true for urine (SCC: 0.96, Supplementary Fig. S2).

**Table 3:** Correlation of metabolites to each other and to standard measurements in CKD patients.

Spearman correlation coefficient		Targeted LC/MS quantification					
		Plasma: Creatinine	Plasma: C-mannosyltryptophan	Plasma: Pseudouridine	Urine: Creatinine	Urine: C-mannosyltryptophan	Urine: Pseudouridine
Targeted LC/MS quantification	Plasma: Creatinine	1					
	Plasma: C-mannosyltryptophan	0.58	1				
	Plasma: Pseudouridine	0.71	0.88	1			
	Urine: Creatinine	0.01*	−0.06	−0.04	1*		
	Urine: C-mannosyltryptophan	−0.21	0.36	0.23	0.94*	1	
	Urine: Pseudouridine	−0.23	0.22	0.19	0.95*	0.69	1
Stand. clinical laboratory measurement	Serum: Creatinine	0.96	0.63	0.74	0.06*	−0.16	−0.23
	Serum: Cystatin C	0.71	0.79	0.85	−0.02*	0.21	0.11
	eGFR (based on serum creatinine)	−0.87	−0.70	−0.77	0.00*	−0.04	0.08
	eGFR (based on cystatin C)	−0.66	−0.79	−0.84	0.02*	−0.28	−0.15
	Urine: Creatinine	0.04	−0.04	−0.04	0.96*	0.89*	0.91*
	Urine: UACR	0.06	0.00	0.04	−0.20*	−0.01	0.10

Units for targeted measurements: plasma - µmol/L, urine - µmol/mmol creatinine or µmol/L (if not standardized for dilution, indicated by *); Units for standard clinical laboratory: serum creatinine: mg/dL, serum cystatin C: mg/L, eGFR: mL/min/1.73 m², UACR: mg/g. UACR: urine albumin-to-creatinine ratio.

Furthermore, we evaluated the associations of C-mannosyltryptophan and pseudouridine with eGFR^5^ using absolute metabolite concentrations to provide estimated changes in eGFR per unit change of metabolite on an interpretable scale. Models including one of the metabolites showed highly significant associations (Supplementary Table S1 and S2). When including both metabolites at the same time, however, only association with pseudouridine remained significant.

### Comparison of non-targeted and targeted measurements

The choice of the QMDiab study for this project allowed us to evaluate the important question whether data from non-targeted measurements are suitable to calculate composite measures used in nephrology, the urinary metabolite-to-creatinine ratio and the metabolite’s FE. The latter is of particular interest, because its calculation requires both serum (plasma) and urine concentrations of creatinine and the metabolite of interest, for which measurements that are not generated using an isotope-labeled standard may not be comparable. We therefore used data from the 110 QMDiab individuals, for whom both targeted and non-targeted measurements were available, to address this second aim.

**Figure 3:**
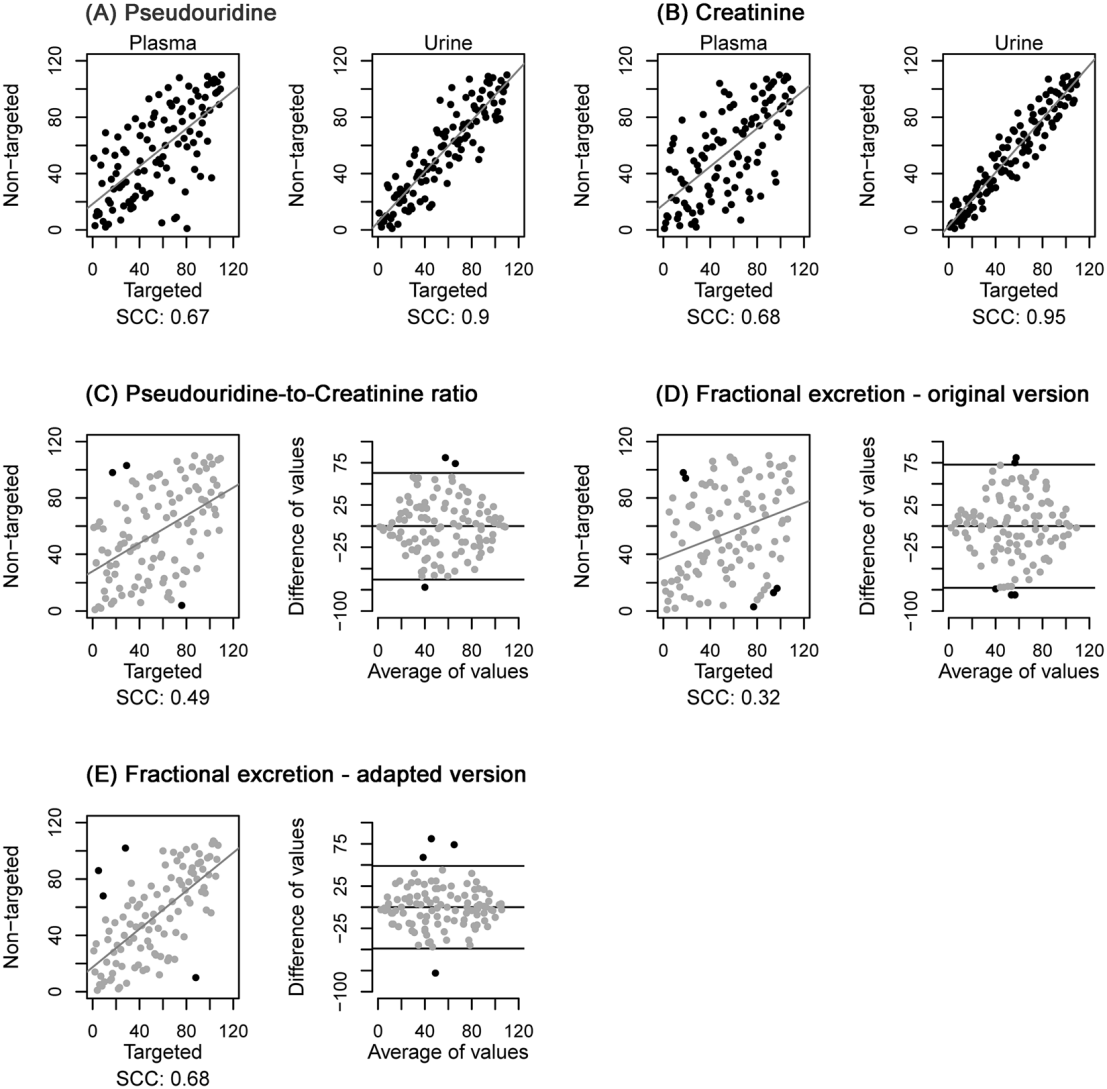
Comparison of ranks of targeted and non-targeted mass spectrometric measurements of pseudouridine and creatinine as well as respective composite measures of pseudouridine in individuals without CKD. (**A**) Pseudouridine, (**B**) Creatinine, (**C**) Urinary pseudouridine-to-creatinine ratio, (**D**) Fractional excretion – original version: using creatinine measurements from respective platform, i.e. targeted or non-targeted, (**E**) Fractional excretion – adapted version: using standard clinical laboratory measurement of creatinine. SCC: Spearman correlation coefficient corresponding to slope of displayed regression line of rank-transformed measurements.

For pseudouridine, targeted and non-targeted measurements in plasma correlated well (SCC: 0.67) and even better in urine (SCC:0.90, Fig. 3A). Similar results were obtained for creatinine (Fig. 3B). However, agreements weakened when combining these measurements into the urinary metabolite-to-creatinine ratio or the FE (Fig. 3C,D). The SCC for the FE of pseudouridine, for example, was only 0.32.

Since creatinine is used both for accounting for urine dilution by calculating the urinary metabolite-to-creatinine ratio as well as in the calculation of FE, accurate quantification of creatinine is of particular importance. We thus investigated correlations with creatinine from a standard clinical laboratory test and found very good agreement with all other measurements of creatinine (SCC:>0.9, Supplementary Fig. S3), except for the non-targeted, nominal mass platform-based measurement of creatinine in plasma (SCC:0.67). We therefore examined whether the use of standard clinical creatinine in the estimation of the FEs would improve the agreement. Indeed, correlations for the FE of pseudouridine improved (SCC: from 0.32 to 0.68, Fig. 3E), suggesting that substitution of non-targeted creatinine with standard clinical creatinine for the calculation of FEs for metabolites can be appropriate when a standard creatinine test is available. Similar observations were also made for C-mannosyltryptophan (Supplementary Fig. S4 **)**.

Although such an approach might be useful, creatinine values from a standard clinical test may not always be available in population studies. While there is no non-creatinine alternative for the calculation of the FE, there are alternatives other than the urinary metabolite-to-creatinine ratio to correct urine metabolite concentrations for dilution. Firstly, osmolality can be quantified as part of a metabolomics study and can be used for this purpose. Secondly, when a larger number of metabolites are quantified simultaneously, individual dilution factors can be deduced using a probabilistic approach, called probabilistic quotient (PQ) normalization^9^. These alternative measures of urine dilution showed good correlation with urinary creatinine from the clinical assay (Supplementary Fig. S5). Thus, osmolality or PQ-normalization might be used to derive dilution-corrected concentrations of metabolites in urine when creatinine from a standard clinical test is not available.

## Discussion

The validation and characterization of non-traditional biomarkers in healthy and diseased individuals is a challenging but necessary step on the way to their clinical use. Our study reports absolute blood plasma and urine concentrations of both C-mannosyltryptophan and pseudouridine^5^ in individuals with and without CKD. Capitalizing on the absolute quantification of pseudouridine and C-mannosyltryptophan in both plasma and urine, our results provide evidence for net re-absorption of both metabolites during tubular passage.

Our study extends findings from previous studies in several ways: first, previous metabolomics studies in nephrology often carried out global screens based on non-targeted metabolite quantification and/or used targeted methods that did not contain isotope-labeled internal standards for all of the metabolites^5–7,10–13^. Here, we specifically focused on two non-traditional kidney function marker candidates that were measured using a targeted technique with isotope-labeled internal standards. Secondly, previous studies of pseudouridine and C-mannosyltryptophan have evaluated their concentrations in blood or urine^14–17^, but they had not compared their concentration ranges in individuals with and without CKD. In addition, the absolute quantification of these kidney function markers in both blood and urine minimizes the influence of matrix effects that can affect the calculation of FE.

The FE of a metabolite is an important measure when searching for renal filtration markers based on comparison with the clearance of exogenous kidney function markers such as inulin. In this setting, an ideal filtration marker should be produced at a constant rate and show neither evidence for net secretion nor reabsorption, i.e., a FE of 100%. Pseudouridine is a modified nucleoside originating from RNA catabolism^18^. Because of its constant production and excretion as well as its free filtration, it has been evaluated as an endogenous renal filtration marker as early as the 1980’s^14^. However, it had been dismissed as a filtration marker because of its partial net reabsorption, consistent with our and other estimates^15^. C-mannosyltryptophan results from N-glycosylation, a post-translational modification, of tryptophan^19^. Takahira and colleagues showed that C-mannosyltryptophan reflected inulin clearance much better than creatinine clearance did^16^. They concluded that C-mannosyltryptophan might be suited to replace inulin as a renal function marker, consistent with other reports demonstrating the superiority of C-mannosyltryptophan as a renal function marker as compared to creatinine^17^. In our study, the FE of C-mannosyltryptophan was 71% in individuals without CKD, similar to that observed for pseudouridine. Together with our previous study showing a high correlation of pseudouridine and C-mannosyltryptophan with measured GFR^5^, these results suggest that an FE close to 100% is not a prerequisite for a filtration marker. Future complementary filtration markers are likely to be identified in an agnostic manner similar to the one used in our previous study^5^.

While the targeted measurements of C-mannosyltryptophan and pseudouridine in this study generally agreed well with the initial non-targeted measurements, agreement for the FE and the urinary metabolite-to-creatinine ratio, composite metabolite measures in nephrology, was lower. Potential explanations are the combined effects of the less than accurate mass spectrometric determination of analytes in the absence of stable isotope-labeled internal standards to correct for differences in the composition of biological matrices that may affect the ionization of the compounds of interest. This most likely explains the higher FE values reported by Solini *et al*. based on their non-targeted quantification of pseudouridine and C-mannosyltryptophan^7^. Although our data suggest that the FE should not be calculated using non-targeted measurements of C-mannosyltryptophan and pseudouridine, this might not be true for all metabolites and could change with future improvements of measurement platforms.

Previous studies have also compared the agreement between non-targeted and targeted mass spectrometric measurements of metabolites^20–25^. Yet *et al*., for instance, compared two of the most frequently used mass spectrometry based metabolomics platforms, the non-targeted Metabolon platform and the Biocrates platform, which uses stable isotope-labeled internal standard for some but not all of its metabolites^25^. The authors found that correlations between metabolites quantified on both platforms varied largely, highlighting that general assumptions about agreements across metabolomics platforms are difficult. Overall, as in transcriptomics and proteomics studies, biomarkers discovered by non-targeted metabolomics methods should be validated prior to their clinical translation, by means of proper targeted methods that allow their accurate and, preferentially, absolute determination.

While this project provides valuable information on C-mannosyltryptophan and pseudouridine, some potential limitations warrant mention: metabolite concentrations determined for control participants of the Qatari QMDiab study may not be globally representative. However, the generally higher concentrations in CKD patients are in line with our previous study examining these differences in a European sample, supporting generalizability at least within ethnic groups^5^.

In summary, accurate quantification of non-traditional biomarkers and their additional characterization in different population samples and biofluids are necessary steps prior to their potential clinical application.

## Methods

### Study populations

#### QMDiab study

The Qatar Metabolomics Study on Diabetes (QMDiab) was conducted in 2012 at the Dermatology Department of Hamad Medical Corporation in collaboration with the Weill Cornell Medical College in Doha, Qatar. This cross-sectional type 2 diabetes case-control study enrolled 374 participants of Arabic, South Asian, Filipino and other ancestry as described previously^26^. The QMDiab study was approved by the Institutional Review Boards of Hamad Medical Corporation (HMC) and Weill and Cornell Medicine – Qatar (WCM-Q) under Research Protocol number 11131/11 and conducted in accordance with relevant guidelines and regulations. After obtaining written informed consent from participants, samples of non-fasting plasma and spot urine were collected, put on dry ice for transport, processed and aliquoted using a standardized protocol and stored at −80 °C within 6 hours^26^. They were not thawed prior to metabolite measurements.

#### GCKD study

The German Chronic Kidney Disease (GCKD) study is a prospective cohort study of patients with CKD treated by nephrologists. It was approved by the ethics committees of the University of Erlangen (data coordinating center) and all other participating regional centers (Aachen, Berlin, Freiburg, Hannover, Heidelberg, Jena, Munich, Würzburg). The study was registered in the national registry for clinical studies (DRKS 00003971) and carried out in accordance with relevant guidelines and regulations. Between 2010 and 2012, 5,217 eligible adult patients provided written consent and were enrolled into the study^27^. All patients had CKD defined as eGFR of 30–60 mL/min/1.73 m^2^ or either urine albumin-to-creatinine ratio (UACR) > 300 mg/g or a protein-to-creatinine ratio >500 mg/g when eGFR was >60 mL/min/1.73 m^2^. Trained personnel obtained information; blood and spot urine were collected and immediately processed within a maximum of two hours (most within 30 minutes). They were shipped on dry ice on the same day to a central laboratory for routine clinical chemistry and to the study’s central biobank where they were immediately stored at −80 °C for future analyses^28^. Samples used in this study were thawed a maximum of two times prior to metabolite measurements. A complete description of the study design and the recruited study population can be found elsewhere^27,29^.

#### Data availability

The data that support the findings of this study are available from the parent studies, but restrictions apply to the availability of these data, which were used under license for the current study, and so are not publicly available. Data are, however, available from the authors upon reasonable request and with permission of the Steering Committees of the parent studies.

### Measurements

In this project, non-targeted and targeted mass spectrometric measurements of C-mannosyltryptophan, pseudouridine and creatinine in plasma and urine were obtained. A comprehensive description can be found in Supplementary Methods S1. Here, briefly: specimens of all QMDiab participants were sent to Metabolon Inc. (Durham, NC) for non-targeted quantifications using GC/MS and LC/MS in July 2012^26^. All targeted quantifications were carried out by high-performance liquid chromatography–electrospray ionization–triple quadrupole mass spectrometry (LC/QQQMS) in the presence of the respective stable isotope-labeled internal standards at the Institute of Functional Genomics in Regensburg.

All measurements underwent stringent quality control. After cleaning, targeted measurements (µmol/L) were available from 111 consenting control participants in QMDiab (i.e. non-diabetic), who did not have CKD, and from a random sample of 382 CKD patients enrolled in the GCKD study. Paired measurements for both plasma and urine were obtained from all assessed control participants and 329 CKD patients. Non-targeted measurements (ion count) were available for 110 of the 111 non-CKD individuals of QMDiab with targeted measurements. Overall, the final datasets were rather complete with respect to measurements (<1% missing values per analyte and study).

Moreover, measurements of creatinine in blood (QMDiab: plasma, GCKD: serum) and urine using standard clinical laboratory methods were available. Serum cystatin C and urinary albumin were also available from CKD patients and osmolality from non-CKD individuals. GFR was estimated using the creatinine- or cystatin C-based Chronic Kidney Disease Epidemiology Collaboration (CKD-EPI) formula^2,3^, and UACR (mg/g) was derived from respective urinary measurements of albumin and creatinine.

### Statistical analyses

The study sizes of the two study populations (non-CKD cohort: N = 111, CKD cohort: N = 382) were sufficient to describe the distribution and variability of the two continuous metabolites, C-mannosyltryptophan and pseudouridine in plasma and in urine (unit: µmol/L) as well as their fractional excretions (FE, unit: %). Urinary measurements were standardized to creatinine measured from the respective platform (urinary metabolite-to-creatinine ratio, unit: µmol/mmol creatinine). Fractional excretion was calculated for each metabolite as

$$100 \% \times ({{\rm{Metabolite}}}_{{\rm{Urine}}}\times {{\rm{Creatinine}}}_{{\rm{Plasma}}})/({{\rm{Metabolite}}}_{{\rm{Plasma}}}\times {{\rm{Creatinine}}}_{{\rm{Urine}}}).$$

Numeric and graphical (boxplot, density plot) presentations were used to describe the distributions of single metabolites and FEs. Correlations between two single numerical measurements were quantified using Spearman correlation coefficient (SCC).

Multivariable linear regression was utilized to assess the associations between measured metabolites in plasma and urine (independent variables) with both creatinine-based eGFR and cystatin C-based eGFR (dependent variables) in CKD patients. Three types of models were considered: (A) models including C-mannosyltryptophan, (B) models including pseudouridine, and (C) models including both C-mannosyltryptophan and pseudouridine. For each type and outcome, models adjusting for an increasing number of covariates were fitted: (1) no further adjustment, (2) adjusted for age and sex, and (3) adjusted for age, sex and 8 additional variables known to be associated with CKD: history of coronary heart disease, diabetes mellitus, anti-hypertensive medication use, body mass index, systolic blood pressure, C-reactive protein, high-density lipoprotein and triglycerides. All continuous variables (outcomes, metabolites, covariates) except age were log-transformed for association analyses. Only individuals with complete information were used for association analysis (N = 317, 96%).

The targeted and non-targeted measurements of C-mannosyltryptophan and pseudouridine were compared in the QMDiab study, with special emphasis on the urinary metabolite-to-creatinine ratio and the FE. For each metabolite measured on different platforms within a specific matrix, SCCs were calculated, linear regression models were fitted and data were graphically presented in scatter plots. Bland-Altman plots were used to assess systematic differences in agreement^30^. Following a non-parametric approach to obtain more robust findings, comparisons were performed using ranks of measurements. In contrast, creatinine measurements (standard clinical laboratory test, targeted and non-targeted quantification) were compared based on original scales, the unit used in routine clinical tests.

*R* software (version 3.2.3, www.r-project.org; R Foundation for Statistical Computing) was used for statistical analyses.

## Electronic supplementary material

Supplementary Information
