# Supplementary material for: From Discovery to Translation: Characterization of C-Mannosyltryptophan and Pseudouridine as Markers of Kidney Function
Source: Sci Rep. 2017 Dec 12;7:17400. doi: 10.1038/s41598-017-17107-5 (PMC5727198; doi:10.1038/s41598-017-17107-5)
Supplement: Supplementary file 1 — Supplementary Information [file 41598_2017_17107_MOESM1_ESM.pdf]

From Discovery to Translation: Characterization of C-Mannosyltryptophan and Pseudouridine as Markers of Kidney Function

P Sekula, K Dettmer, FC Vogl, W Gronwald, L Ellmann, RP Mohney, K-U Eckardt, K Suhre, G Kastenmüller, PJ Oefner, A Köttgen

Supplementary Information

CONTENTS

List of GCKD Study Investigators

Methods S1: Description of metabolite measurements and quality control

Table S1: Association of metabolites in plasma with creatinine-based eGFR in CKD patients
Table S2: Association of metabolites in plasma with cystatin C-based eGFR in CKD patients
Table S3: Information on non-targeted metabolites measured by Metabolon
Table S4: Information on targeted measurements
Table S5: Intra- and inter-variability of reference samples for targeted measurements

Figure S1: Densities for targeted measurements for C-mannosyltryptophan and pseudouridine in individuals without and with CKD
Figure S2: Comparison of creatinine measurements using standard clinical laboratory test versus targeted quantification in CKD patients
Figure S3: Comparison of creatinine measurements using different techniques in individuals without CKD
Figure S4: Comparison of ranks of targeted and non-targeted measurements for C-mannosyltryptophan and related measures in individuals without CKD
Figure S5: Correlation between urinary creatinine from standard clinical laboratory and alternative measures of urine dilution in individuals without CKD

References

List of GCKD Study Investigators

University of Erlangen-Nürnberg: Kai-Uwe Eckardt, Heike Meiselbach, Markus Schneider, Thomas Dienemann, Hans-Ulrich Prokosch, Barbara Bärthlein, André Reis, Arif B. Ekici, Susanne Avendaño, Dinah Becker-Grosspitsch, Birgit Hausknecht, Rita Zitzmann, Anke Weigel, Andreas Beck, Thomas Ganslandt, Sabine Knispel

University of Freiburg: Gerd Walz, Anna Köttgen, Ulla Schultheiß, Simone Meder, Erna Mitsch, Ursula Reinhard

RWTH Aachen University: Jürgen Floege, Georg Schlieper, Turgay Saritas, Sabine Ernst, Astrid Blasius, Kerstin Schmitz

Charité, University Medicine Berlin: Elke Schaeffner, Seema Baid-Agrawal, Kerstin Theisen

Hannover Medical School: Hermann Haller, Jan Menne, Elisabeth Bahlmann

University of Heidelberg: Martin Zeier, Claudia Sommerer, Claudia Föllinger

University of Jena: Gunter Wolf, Martin Busch, Rainer Fuß

Ludwig-Maximilians University of München: Thomas Sitter, Claudia Blank

University of Würzburg: Christoph Wanner, Vera Krane, Karina Schönowsky, Antje Börner-Klein

Medical University of Innsbruck, Division of Genetic Epidemiology: Florian Kronenberg, Julia Raschenberger, Barbara Kollerits, Lukas Forer, Sebastian Schönherr, Hansi Weissensteiner

University of Regensburg, Institute of Functional Genomics: Peter Oefner, Wolfram Gronwald, Helena Zacharias

Department of Medical Biometry, Informatics and Epidemiology (IMBIE), University of Bonn: Matthias Schmid, Jennifer Nadal.

A list of nephrologists currently collaborating with the GCKD study is available at http://www.gckd.org.

Methods S1: Description of metabolite measurements and quality control

(1) Non-targeted quantifications

Plasma and urine specimens of all QMDiab participants were sent to Metabolon Inc. (Durham, NC) for non-targeted quantifications using GC/MS and LC/MS in July 2012.[1] Methodological details of measurement techniques are provided elsewhere and below.[2, 3]

Samples were prepared for analysis using the automated MicroLab STAR® system from Hamilton Company. After thawing, 400 ml of extraction solvent, namely methanol containing recovery standards, was added to each 100 ml of serum samples in a 96-well plate format. Recovery standards are carefully chosen so as not to interfere with the measurement of the endogenous compounds. Extraction was carried out by shaking for 2 minutes. After centrifugation, the supernatant of the extract was divided into equal fractions: two for LC/MS (positive and negative ion mode), one for GC/MS analysis, and one as a reserve. Samples were placed briefly on a TurboVap® (Zymark) to remove the organic solvent and were dried under vacuum overnight. Extracts were reconstituted in acidic conditions (0.1% formic acid) for LC/MS in positive ion mode. Analogously, extracts were reconstituted in basic conditions (6.5mM ammonium bicarbonate) for LC/MS in negative ion mode. Both reconstitution solvents contained also 11 internal standards at fixed concentrations. The GC/MS aliquots were derivatized for 1 hour at 60°C with N,O-bistrimethylsilyl-trifluoroacetamide in a solvent mixture.

LC/MS analysis was based on a Waters ACQUITY UPLC and a Thermo-Finnigan LTQ mass spectrometer which consisted of an electrospray ionization (ESI) source and linear ion-trap (LIT) mass analyzer. Two separate columns (2.13100 mm, BEH C18 1.7 mm particle; Waters Corporation) were used for acidic (solvent A: 0.1% formic acid in H_2O; solvent B: 0.1% formic acid in methanol) and basic (solvent A: 6.5 mM ammonium bicarbonate aqueous solution, pH 8.0; and solvent B: 6.5 mM ammonium bicarbonate in 98% methanol) mobile phase conditions, optimized for positive and negative ESI, respectively. After injection of the sample extracts, the columns were developed in a gradient of 100% A to 98% B in 11-minute runtime at a 350 ml/min flow rate. The MS analysis alternated between MS and data-dependent MS^2 scans using dynamic exclusion.

GC/MS analysis, was performed on a Thermo-Finnigan Trace DSQ fast-scanning single-quadrupole mass spectrometer using electron impact ionization. The GC column was 5% phenyl and the column temperature was ramped from 40° to 300° C in 16 min. The instrument was tuned and calibrated for mass resolution and mass accuracy on a daily basis.

The data extraction of the raw mass spec data files yielded information that could be loaded into a relational database and further evaluated. Peaks were detected and quantified from raw mass spec data files using Metabolon's proprietary peak detection and integration software. Compounds were identified by comparison of peak characteristics, namely retention index, mass-to-charge ratio (m/z), and fragmentation spectra to library entries of purified standards or recurrent unknown entities. Identification of known chemical entities was based on comparison to metabolomic library entries of

purified standards. Additional entities could be identified by virtue of their recurrent nature (both chromatographic and mass spectral). These compounds have the potential to be identified by future acquisition of a matching purified standard or by classical structural analysis. Raw data are provided in ion counts.

Instrument variability was determined by calculating the median relative standard deviation for the internal standards. For purposes of further quality assurance and quality control (QA/QC), a number of additional samples were included with each day's analysis: (i) reference human plasma samples, (ii) pool samples created by taking a small aliquot from every study sample, (iii) process blanks (water and solvent samples). These QC samples are primarily used to evaluate the process stability for each study as well as aiding in the data curation. The median coefficient of variation for all endogenous metabolites present in 100% of the technical replicates of study pool samples was 13% in plasma and 9% in urine.

(2) Targeted LC/MS quantifications

Targeted quantification of C-mannosyltryptophan, pseudouridine and creatinine in plasma and urine was carried out by high-performance liquid chromatography–electrospray ionization–triple quadrupole mass spectrometry (LC/QQQMS) in the presence of the respective stable isotope-labeled internal standards at the Institute of Functional Genomics in Regensburg.

Urine specimens were pre-diluted 1:5 with water (PURELAB Plus water). Then, 10 µL of internal standard mix (containing creatinine-d3, pseudouridine-^{13}C, $^{15}N_2$, and C-mannosyltryptophan-d4 at a concentration of 50 µM each in 0.1% formic acid in water) were added to 10 µL of pre-diluted urine. The sample was then diluted to a final volume of 100 µL with water.

For analysis of plasma, 10 µL of internal standard mix was added to 50 µL of plasma followed by 200 µL methanol. The sample was vortexed and then centrifuged at 13,800×g at 4 °C for 5 min. The supernatant was collected and dried using a vacuum evaporator (CombiDancer, Hettich AG,Bäch, Switzerland). The residue was re-dissolved in 100 µL of water.

Targeted LC/MS analysis was carried out using an Agilent 1200 SL HPLC system (Böblingen, Germany) hyphenated to a 4000 QTrap mass spectrometer with a TurboV electrospray ion source (Sciex, Darmstadt, Germany). Detection was performed with positive mode ionization and multiple reaction monitoring. HPLC separation was carried out using an Atlantis T3 (2.1×150-mi.d., 3 µm, Waters, Eschborn, Germany) reversed-phase column equipped with a C18 security guard column (Phenomenex, Aschaffenburg, Germany) and gradient elution with mobile phases A (0.1% formic acid in water, v/v) and mobile phase B (0.1% formic acid in acetonitrile, v/v). The column was kept at 30°C and 10 µL of sample was injected. Samples were analyzed in random order with reference samples, calibration check samples and blanks inserted after 20 samples. Quantification was performed using calibration curves with the corresponding stable isotope-labeled analog as internal standard. Data analysis was performed using Analyst version 1.6.2 (Sciex).

(3) Quality control

All non-targeted and targeted measurements on C-mannosyltryptophan, pseudouridine and creatinine underwent stringent quality control.

Non-targeted measurements (ion count) were available from 170 control participants of QMDiab (i.e. non-diabetic) and median-scaled by run day to account for inter-day differences. The quality of measurements assessed by coefficient of variation was below 20% for all metabolites (**Table S3**). Further quality control led to the exclusion of all measurements for two individuals because of an excess of outlying measurements defined as measurements greater or smaller than mean±4×standard deviation. Otherwise, only few outlying values in the remaining dataset were detected and removed from the dataset.

Targeted quantifications (µmol/L) in plasma or urine were available from 111 consenting control participants in QMDiab who did not have CKD, and from a random sample of 382 CKD patients enrolled in the GCKD study. Paired measurements from both plasma and urine were obtained from all assessed control participants and 329 CKD patients. Ranges of metabolite quantification and measures of quality (accuracy, coefficients of variations) per study population as well as batch-related intra-/inter-variability in reference measurements are provided in **Tables S4 and S5**. Per study population and metabolite, accuracy of measurements was in the range of 80% to 120%. All measurements were within the range of quantification. Coefficients of variation were <20% for all measurements except for pseudouridine in plasma of GCKD participants (20.8%). Very few outlying measurements were detected and removed from the dataset.

Overall, the final data sets were rather complete with respect to measurements (<1% missing values per analyte and study). Non-targeted measurements were available for 110 of the 111 non-CKD individuals of QMDiab with targeted measurements.

(4) Further measurements

Several measurements of creatinine using standard clinical laboratory methods were available for this project. In the QMDiab study, creatinine in plasma was measured in the context of the usual assessment of clinical chemistry of the clinic. Creatinine in urine was measured by Chenomx Inc. (Edmonton, Canada) using nuclear magnetic resonance. In the GCKD study, creatinine in serum and in urine was measured in a central laboratory using an IDMS traceable assay (Creatinine plus, Roche). Furthermore, Cystatin C in serum and urinary albumin were measured using the Tina-quant assay (Roche) and Tina-quant Albumin Modular (Roche), respectively, in CKD patients and UACR (in mg/g) was derived from respective urinary measurements of albumin and creatinine. Information on osmolality was available in the QMDiab study that was measured by Metabolon Inc. (Durham, NC) at the same time as non-targeted metabolites were quantified.

For both studies, GFR was estimated using the creatinine-based Chronic Kidney Disease Epidemiology Collaboration (CKD-EPI) formula.[4] In addition, cystatin C-based eGFR was calculated for CKD patients.[5]

Table S1: Association of metabolites in plasma with serum creatinine-based eGFR[1] in CKD patients

Model	Model A: C-mannosyltryptophan[1] (targeted LC/MS)			Model B: Pseudouridine[1] (targeted LC/MS)			Model C: C-mannosyltryptophan[1] and pseudouridine[1]			
	Beta (SE)	P value[2]	Adj. R^2	Beta (SE)	P value[2]	Adj. R^2		Beta (SE)	P value[2]	Adj. R^2
1) Metabolite only	-0.61 (0.03)	**2.9e-53**	0.53	-0.80 (0.04)	**3.6e-68**	0.62	CM:	-0.08 (0.07)	2.0e-01	0.62
							PU:	-0.71 (0.08)	**6.7e-17**	
2) Metabolite + age, sex	-0.58 (0.03)	**1.0e-49**	0.55	-0.78 (0.03)	**8.5e-68**	0.66	CM:	-0.004 (0.07)	9.5e-01	0.66
							PU:	-0.77 (0.08)	**9.8e-20**	
3) Metabolite + age, sex, 8 other[3]	-0.59 (0.03)	**2.9e-47**	0.56	-0.78 (0.04)	**2.9e-63**	0.66	CM:	-0.03 (0.07)	6.6e-01	0.66
							PU:	-0.74 (0.08)	**1.1e-17**	

Note that only individuals with complete information for all models considered were included (N=317). Measurements for serum creatinine and serum cystatin C in GCKD were obtained with standard clinical laboratory test. Adj. R^2: adjusted R^2; LogLik: log-likelihood; -2LogL: log-likelihood ratio statistic

[1] log-transformed; [2] Wald test (H_0: Beta = 0), bold marking significant associations (p-value < 0.05/12=4.2e-03); [3] further adjustment factors: history of coronary heart disease, diabetes, medication use reducing blood pressure, body mass index, systolic blood pressure, C-reactive protein, high-density lipoprotein, triglycerides (log-transformation applied to continuous variables)

For ease of interpretation: considering the log-transformations, the regression coefficient of C-mannosyltryptophan in model A1 of -0.61, for example, can be interpreted as an 0.61% lower average eGFR per 1% higher C-mannosyltryptophan.

Table S2: Association of metabolites in plasma with serum cystatin C-based eGFR[1] in CKD patients

	Model A: C-mannosyltryptophan[1] (targeted LC/MS)			Model B: Pseudouridine[1] (targeted LC/MS)			Model C: C-mannosyltryptophan (CM)[1] and pseudouridine (PU)[1]			
**Model**	**Beta (SE)**	**P value[2]**	**Adj. R²**	**Beta (SE)**	**P value[2]**	**Adj. R²**		**Beta (SE)**	**P value[2]**	**Adj. R²**
1) Metabolite only	-0.70 (0.03)	**3.9e-69**	0.62	-0.89 (0.03)	**2.0e-81**	0.69	CM:	-0.21 (0.06)	**7.2e-04**	0.70
							PU:	-0.66 (0.08)	**1.9e-16**	
2) Metabolite + age, sex	-0.67 (0.03)	**2.2e-65**	0.63	-0.87 (0.03)	**8.4e-82**	0.71	CM:	-0.14 (0.06)	2.8e-02	0.72
							PU:	-0.72 (0.08)	**3.9e-19**	
3) Metabolite + age, sex, 8 other[3]	-0.68 (0.03)	**5.4e-61**	0.64	-0.87 (0.03)	**1.8e-76**	0.71	CM:	-0.14 (0.07)	3.2e-02	0.72
							PU:	-0.72 (0.08)	**3.8e-18**	

Note that only individuals with complete information for all models considered were included (N=317). Measurements for serum creatinine and serum cystatin C in GCKD were obtained with standard clinical laboratory test. Adj. R²: adjusted R²; LogLik: log-likelihood; -2LogL: log-likelihood ratio statistic

[1] log-transformed; [2] Wald test (H_0: Beta = 0), bold marking significant associations (p-value < 0.05/12=4.2e-03); [3] further adjustment factors: history of coronary heart disease, diabetes, medication use reducing blood pressure, body mass index, systolic blood pressure, C-reactive protein, high-density lipoprotein, triglycerides (log-transformation applied to continuous variables)

For ease of interpretation: considering the log-transformations, the regression coefficient of C-mannosyltryptophan in model A1 of -0.70, for example, can be interpreted as an 0.7% lower average eGFR per 1% higher C-mannosyltryptophan.

Table S3: Information on non-targeted metabolites measured by Metabolon

**Biochemical**	**Creatinine**	**C-mannosyltryptophan**	**Pseudouridine**
Comp ID	513	32675	33442
Super pathway	Amino acid	Amino acid	Nucleotide
Sub pathway	Creatine metabolism	Tryptophan metabolism	Pyrimidine metabolism, uracil containing
PUBCHEM	588		
CAS	60-27-5		1445-07-4
KEGG	C00791		C02067
HMDB-ID	HMDB00562		HMDB00767
Platform	LC/MS	LC/MS	LC/MS
Coefficient of variation			
- Plasma	10.3	10.2	15.4
- Urine	9.6	4.8	6.1

Table S4: Information on targeted measurements

	Plasma				Urine			
	**Mode**	**Accuracy**	**CV**	**ROQ***	**Mode**	**Accuracy**	**CV**	**ROQ***
**QMDiab**								
Creatinine	+	98.5%	1.4	0.055-900	+	98.3%	2.2	1.375-22,500
C-mannosyltryptophan	+	94.4%	2.4	0.0274-450	+	93.8%	1.9	0.685-11,250
Pseudouridine	+	99.2%	6.9	0.055-900	+	114.1%	4.5	1.375-22,500
**GCKD**								
Creatinine	+	95.5%	5.4	0.0658-1,080	+	88.7%	3.6	3.295-27,000
C-mannosyltryptophan	+	95.8%	11.8	0.0440-360	+	97.3%	7.7	0.2745-9,000
Pseudouridine	+	86.9%	20.8	0.0109-179.2	+	89.6%	10.0	0.1365-1,120

* unit: µmol/L (transformed limits: for plasma: *2/1000, for urine: *50/1000)
CV: coefficient of variation; ROQ: range of quantification

Table S5: Intra- and inter-variability of reference samples for targeted measurements

Relative Standard Deviation (RSD)	Batch	Creatinine	Pseudo-uridine	C-mannosyl-tryptophan
**QMDiab**				
Urine reference	1	1,36	8,36	5,78
	2	1,86	7,06	2,63
	3	-*	-*	-*
	Overall†	2,41	7,03	6,02
Serum reference	1	1,66	3,11	6,79
	2	-*	-*	-*
	3	2,28	7,15	-
	Overall†	1,7	7,2	15,27
**GCKD**				
Urine reference, male	1	1,1	11,2	4,7
	2	-*	-*	-*
	3	3,9	9	2,3
	4	3,7	8,6	3,8
	5	2	5,8	2,8
	6	-*	-*	-*
	Overall†	3,4	8,3	7,8
Urine reference, female	1	2,4	2,3	1,7
	2	-*	-*	-*
	3	3,5	6,1	6,9
	4	6,4	8,5	8,3
	5	1,8	8,2	3,6
	6	-*	-*	-*
	Overall†	3,9	8,7	8,9
Plasma reference	1	7,4	14	-‡
	2	4,5	10,9	-‡
	3	3,4	12,1	-‡
	4	1,7	4,5	-‡
	5	-*	-*	-‡
	Overall†	7	18,5	-‡

Overall, measurements were done on 6 plates in GCKD and on 3 plates in QMDiab.

* Batch variability is not presented for plates with <3 reference measurements.

† RSD calculation based on all reference measurements

‡ No reference values for C-mannosyltryptophan in plasma available as the measurements were at the limit of quantification.

Figure S1: Densities for targeted measurements for C-mannosyltryptophan and pseudouridine in individuals without and with CKD

(A) Plasma (µmol/L)

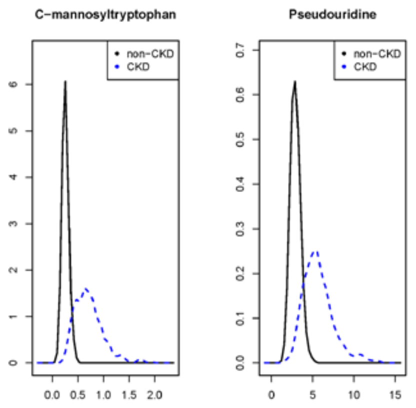

Skewness[1]	C-mannosyl-tryptophan		Pseudouridine	
Scale[2]	Original	Log	Original	Log
Non-CKD	0.79	0.19	0.67	0.19
CKD	1.12	-0.04	1.03	-0.02

(B) Urine (µmol/mmol creatinine)

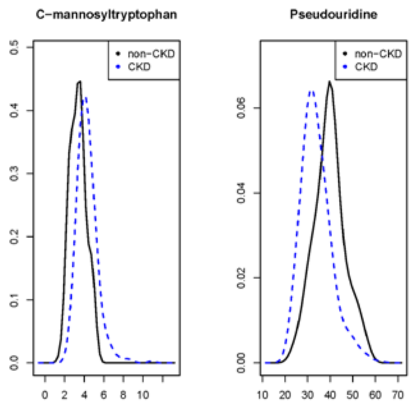

Skewness[1]	C-mannosyl-tryptophan		Pseudouridine	
Scale[2]	Original	Log	Original	Log
Non-CKD	0.26	-0.22	0.16	-0.34
CKD	1.5	0.32	0.95	0.36

(C) Fractional excretion (%)

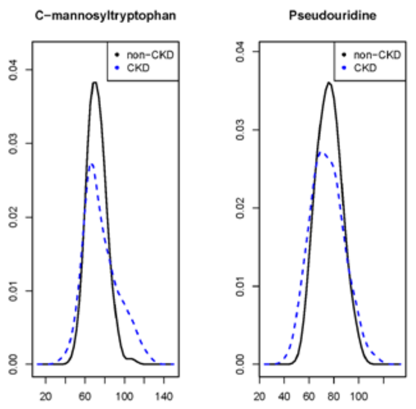

Skewness[1]	C-mannosyl-tryptophan		Pseudouridine	
Scale[2]	original	Log	original	Log
Non-CKD	0.56	0.11	0.19	-0.09
CKD	0.61	0.03	0.28	-0.20

[1] Skewness parameter as measure of symmetry (0 = symmetric distribution); [2] Scale: original scale as presented in plots or natural logarithm transformed . The latter improves skewness except for urinary pseudouridine in individuals without CKD.

Figure S2: Comparison of creatinine measurements using standard clinical laboratory test versus targeted quantification in CKD patients

(A) Plasma/Serum

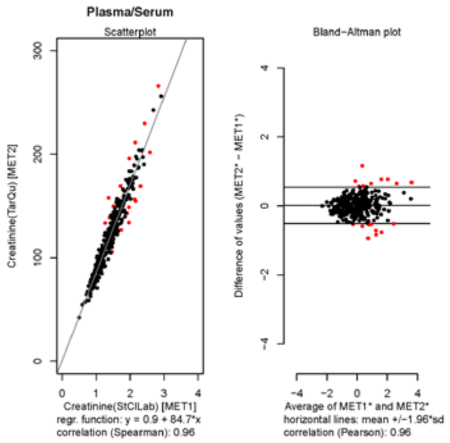

(B) Urine

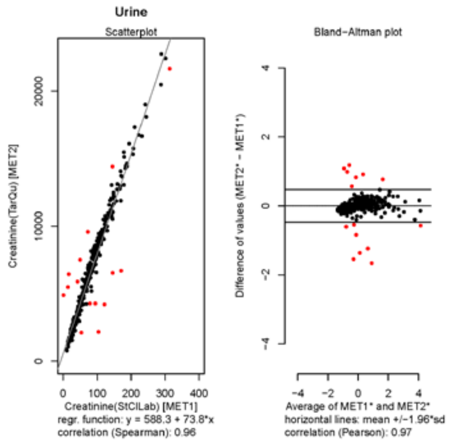

In GCKD, standard clinical laboratory creatinine was measured in serum.

StClLab: standard clinical laboratory; TarQu: targeted LC/MS quantification
* For convenience, normalized values ([meas-mean]/sd) are used for Bland-Altman plot while measurements on original scale are shown in scatter plots (StClLab: mg/dL, TarQu: µmol/L).

Figure S3: Comparison of creatinine measurements using different techniques in individuals without CKD

Plasma **Urine**

(A) Standard clinical laboratory measurement versus targeted quantification

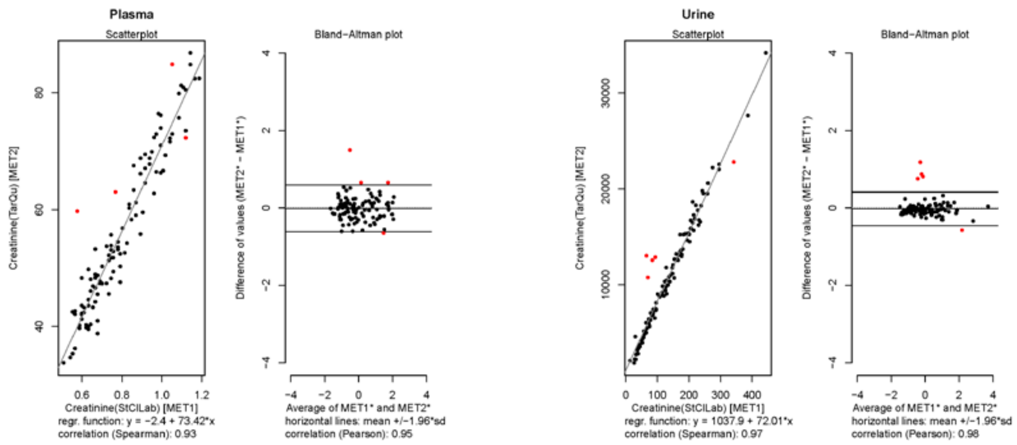

(B) Standard clinical laboratory measurement versus non-targeted quantification

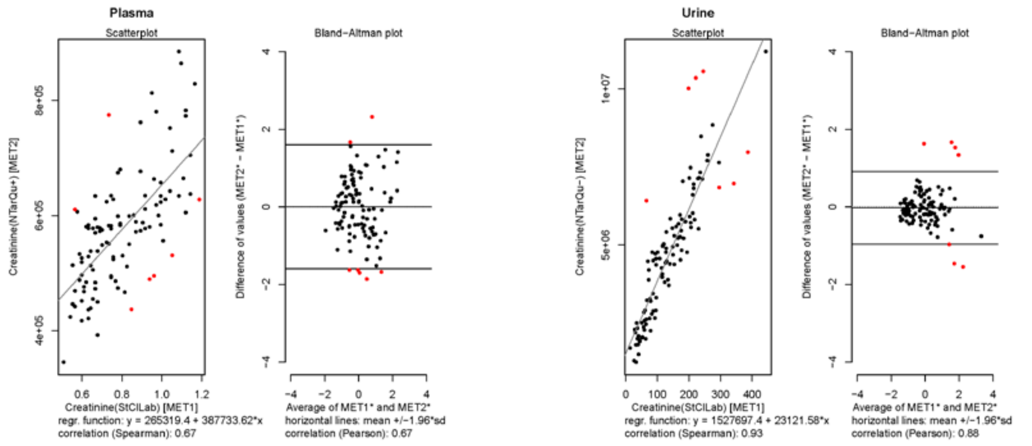

(C) targeted quantification versus non-targeted quantification

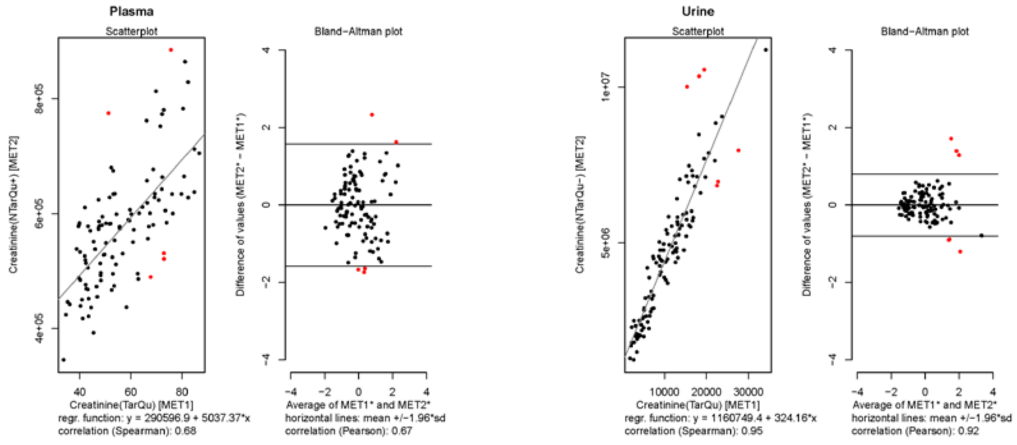

StClLab: standard clinical laboratory; TarQu: targeted LC/MS quantification, NTarQu: non-targeted quantification
* For convenience, normalized values ([meas-mean]/sd) are used for Bland-Altman plot while measurements on original scale are shown in scatter plots (StClLab: mg/dL, TarQu: µmol/L, nTarQu: ion counts).

Figure S4: Comparison of ranks of targeted and non-targeted measurements for C-mannosyltryptophan and related measures in individuals without CKD

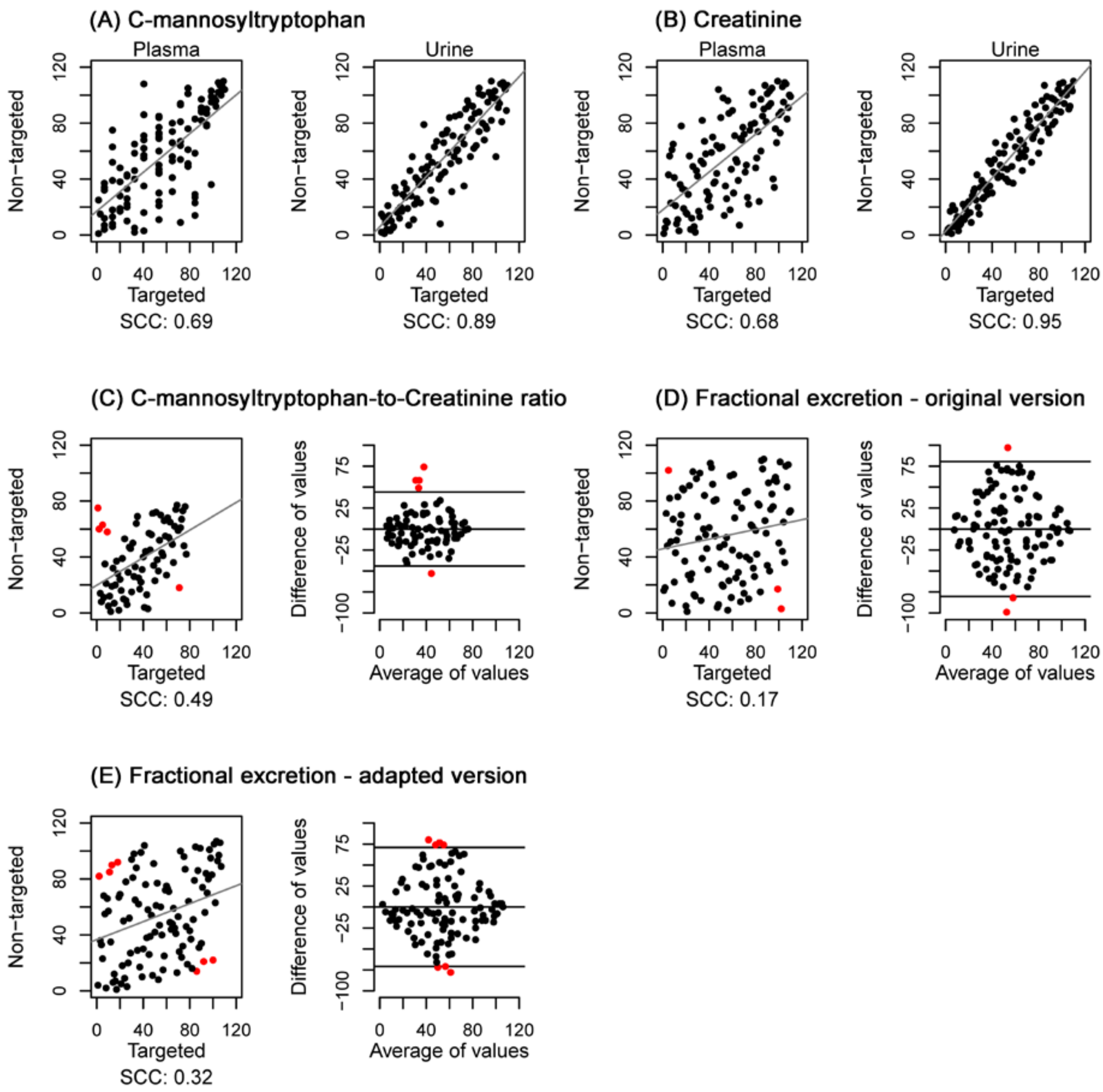

Fractional excretion – original version: For calculation, creatinine measurements from the respective platform, i.e. targeted or non-targeted, were used; Fractional excretion – adapted version: For calculation, standard clinical laboratory measurements of creatinine were used instead of the creatinine measurements from the respective platform.

Correlation (Spearman correlation coefficient) corresponds to slope of displayed regression line of rank-transformed measurements.

Figure S5: Correlation between urinary creatinine from standard clinical laboratory and alternative measures of urine dilution in individuals without CKD

Creatinine (StClLab) vs Osmolality

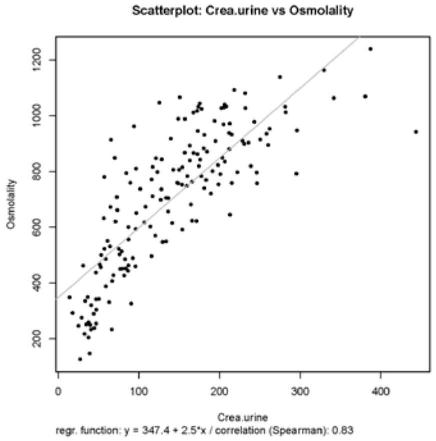

Creatinine (StClLab) vs PQ-normalization

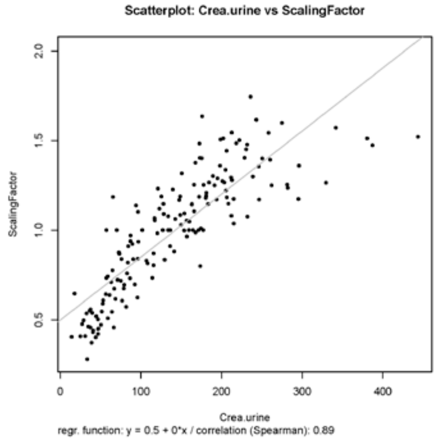

StClLab: standard clinical laboratory
Units: creatinine (standard): mg/dL, Scaling Factor (PQ-normalization): -, Osmolality: -.
Note: all individuals with information on osmolality and urinary creatinine (StClLab) were used for this presentation (N=166).

References

1. Mook-Kanamori, DO, Selim, MM, Takiddin, AH, Al-Homsi, H, Al-Mahmoud, KA, Al-Obaidli, A, Zirie, MA, Rowe, J, Yousri, NA, Karoly, ED, Kocher, T, Sekkal Gherbi, W, Chidiac, OM, Mook-Kanamori, MJ, Abdul Kader, S, Al Muftah, WA, McKeon, C, Suhre, K: 1,5-Anhydroglucitol in saliva is a noninvasive marker of short-term glycemic control. *J Clin Endocrinol Metab,* 99**:** E479-483, 2014.
2. Evans, AM, DeHaven, CD, Barrett, T, Mitchell, M, Milgram, E: Integrated, nontargeted ultrahigh performance liquid chromatography/electrospray ionization tandem mass spectrometry platform for the identification and relative quantification of the small-molecule complement of biological systems. *Anal Chem,* 81**:** 6656-6667, 2009.
3. Dehaven, CD, Evans, AM, Dai, H, Lawton, KA: Organization of GC/MS and LC/MS metabolomics data into chemical libraries. *J Cheminform,* 2**:** 9, 2010.
4. Levey, AS, Stevens, LA, Schmid, CH, Zhang, YL, Castro, AF, 3rd, Feldman, HI, Kusek, JW, Eggers, P, Van Lente, F, Greene, T, Coresh, J, Ckd, EPI: A new equation to estimate glomerular filtration rate. *Ann Intern Med,* 150**:** 604-612, 2009.
5. Inker, LA, Schmid, CH, Tighiouart, H, Eckfeldt, JH, Feldman, HI, Greene, T, Kusek, JW, Manzi, J, Van Lente, F, Zhang, YL, Coresh, J, Levey, AS, Investigators, C-E: Estimating glomerular filtration rate from serum creatinine and cystatin C. *N Engl J Med,* 367**:** 20-29, 2012.
